# On the Use of TMS to Investigate the Pathophysiology of Neurodegenerative Diseases

**DOI:** 10.3389/fneur.2020.584664

**Published:** 2020-11-03

**Authors:** Vishal Rawji, Anna Latorre, Nikhil Sharma, John C. Rothwell, Lorenzo Rocchi

**Affiliations:** Department of Clinical and Movement Neurosciences, UCL Queen Square Institute of Neurology, University College London, London, United Kingdom

**Keywords:** transcrancial magnetic stimulation (TMS), neurodegenarative diseases, motor neuron disease (MND), amyotrophic lateral sclerosis, dementia, Alzheimer's disease, dementia with lewy bodies (DLB), Parkinson's disease

## Abstract

Neurodegenerative diseases are a collection of disorders that result in the progressive degeneration and death of neurons. They are clinically heterogenous and can present as deficits in movement, cognition, executive function, memory, visuospatial awareness and language. Transcranial magnetic stimulation (TMS) is a non-invasive brain stimulation tool that allows for the assessment of cortical function *in vivo*. We review how TMS has been used for the investigation of three neurodegenerative diseases that differ in their neuroanatomical axes: (1) Motor cortex—corticospinal tract (motor neuron diseases), (2) Non-motor cortical areas (dementias), and (3) Subcortical structures (parkinsonisms). We also make four recommendations that we hope will benefit the use of TMS in neurodegenerative diseases. Firstly, TMS has traditionally been limited by the lack of an objective output and so has been confined to stimulation of the motor cortex; this limitation can be overcome by the use of concurrent neuroimaging methods such as EEG. Given that neurodegenerative diseases progress over time, TMS measures should aim to track longitudinal changes, especially when the aim of the study is to look at disease progression and symptomatology. The lack of gold-standard diagnostic confirmation undermines the validity of findings in clinical populations. Consequently, diagnostic certainty should be maximized through a variety of methods including multiple, independent clinical assessments, imaging and fluids biomarkers, and post-mortem pathological confirmation where possible. There is great interest in understanding the mechanisms by which symptoms arise in neurodegenerative disorders. However, TMS assessments in patients are usually carried out during resting conditions, when the brain network engaged during these symptoms is not expressed. Rather, a context-appropriate form of TMS would be more suitable in probing the physiology driving clinical symptoms. In all, we hope that the recommendations made here will help to further understand the pathophysiology of neurodegenerative diseases.

## Introduction

Neurodegenerative diseases constitute a heterogeneous group of disorders of the central nervous system characterized by gradual loss of structure and function of neurons, which eventually results in their death. This progressive damage leads to the development of a plethora of different signs and symptoms, based on the neural structures involved. Examples of neurodegenerative disorders include Parkinson's disease, Alzheimer's disease, and motor neuron disease. Whilst cell-based literature has informed about molecular mechanisms of disease (1), and bioinformatic approaches have informed of population-level exposure-disease risk associations (2), they do not explain physiological changes caused by neurodegenerative diseases in humans.

Transcranial magnetic stimulation (TMS) is a non-invasive brain stimulation technique, which uses a time-varying magnetic field to induce electrical currents in cortical areas of interest; these currents ultimately cause neuronal depolarization and action potential generation. Using TMS in humans allows the assessment of cortical function *in vivo* and can inform about the network characteristics of pathological scenarios, such as neurodegenerative diseases. This article reviews current literature to exemplify how TMS has been used to inform about neurodegenerative diseases. We focus on diseases affecting three different neuroanatomical axes to highlight the strengths and weaknesses of TMS:

1. Motor cortex—corticospinal tract (motor neuron diseases)
2. Non-motor cortical areas (dementias)
3. Subcortical structures (parkinsonisms)

By evaluating the strengths and weaknesses of TMS through this review, we offer a series of recommendations to the non-invasive brain stimulation community that will hopefully contribute to further development of the use of TMS in neurodegenerative diseases.

## Transcranial Magnetic Stimulation

TMS is a non-invasive brain stimulation tool, which can specifically activate targeted brain regions. It is painless, well-tolerated and allows investigators to probe the cerebral cortex in humans. The utility of TMS has traditionally been dependent on the ability to record the output, and much of the understanding of the effects of TMS on cortical circuits comes from stimulation of the primary motor cortex (M1). If applied over M1 at a suprathreshold stimulus intensity, TMS results in muscular contractions in contralateral muscles, in a somatotopically organized manner, which can be measured as electromyographic (EMG) activity using surface electrodes. The resultant output is called the motor evoked potential (MEP) and the ease with which MEPs can be produced using TMS is termed corticospinal excitability (CSE), as the major output stimulated using TMS is *via* the corticospinal tract (CST). The amplitude of the MEP varies as a function of the CSE at the time of stimulation. For example, CSE is increased if a subject expects that that the corresponding muscle will be activated and decreases if it is likely that the muscle will not be called into action (3).

Contrary to popular notions, CSE is not solely a measure of cortical excitability; the MEP is derived from cortical, spinal, nerve root, peripheral nerve, neuromuscular junction and muscle inputs (4–7). The cortical contribution to the MEP comes in the form of descending corticospinal volleys (8) generated after stimulation of cortical interneurons that synapse onto layer V CST neurons. These descending volleys summate upon spinal motor neurons and generate the MEP.

### TMS as a Tool to Investigate Motor Cortex Physiology

The aim of this section is to give a brief account of the mechanisms underlying the TMS applications mentioned throughout the manuscript. Broadly, they can be categorized into four groups: single-pulse TMS (spTMS), paired-pulse TMS (ppTMS), connectivity measures, and repetitive TMS (rTMS).

As mentioned in the previous section, spTMS applied to M1 can be used to probe CSE through several measures derived from MEP. One such measure is the “threshold,” i.e., the stimulus intensity required to evoke an MEP of a pre-determined amplitude (usually between 50 and 200 μV); this can be probed at rest (resting motor threshold—RMT) and during muscle contraction (active motor threshold—AMT) (9). The peak-to-peak amplitude of MEPs also gives an indication of CSE and scales as a function of various cognitive processes such as effort (10), motor preparation (3, 11, 12), and surprise (13). As a more accurate measure of CSE, it is possible to test the input-output (I/O) relationship between increasing TMS intensity and resulting MEP amplitude, thus estimating the gain of M1 (14). spTMS can also be used to assess M1 inhibitory mechanisms, putatively relying on GABA_B_-mediated neurotransmission, by measuring the cortical silent period (CSP) [i.e., the interruption of voluntary muscle contraction caused by a single TMS pulse (15, 16)]. Besides CSE, spTMS can also give information about integrity of the CST by assessing the central motor conduction time (CMCT) [i.e., the time taken for descending volleys to travel from M1 to anterior horn cells in the spinal cord (17)].

One shortcoming of spTMS is that MEPs obtained by single pulses give information only about the global excitability of CST neurons and associated intracortical circuitry. One way to overcome this limitation is by means of ppTMS, with stimuli applied through the same coil. By varying the interstimulus interval (ISI) between pulses, as well as the intensity of the first, conditioning stimulus, it is possible to probe different inhibitory or excitatory intracortical circuits. Examples of ppTMS paradigms are short intracortical inhibition (SICI), intracortical facilitation (ICF), and long intracortical inhibition (LICI), which are thought to probe GABA_A_-mediated inhibition (14, 18), glutamatergic facilitation (18, 19), and GABAB-mediated inhibition (20, 21), respectively. A particular form of interaction, called short intracortical facilitation (SICF), can be obtained when the test pulse is followed, rather than preceded, by a conditioning pulses, with an ISI compatible with the latency difference between the first and the following descending wavelets induced by TMS (22, 23).

While TMS is often considered a focal source of stimulation, it is recognized that it may induce widespread activation *via* large-scale cortical networks. This principle can be used to assess functional connectivity by twin-coil TMS, where a conditioning pulse is delivered to a region of interest (ROI) and is followed by M1-TMS. With this approach, connectivity has been probed between M1 and a large number of ROIs, including ipsilateral (24, 25) and contralateral (26, 27) nodes of the motor network, as well as the contralateral cerebellar hemisphere (28, 29). M1 connectivity can also be probed with activation of sensory pathways by modality-specific stimuli. The most common approach is to pair electrical stimulation of a peripheral nerve with M1-TMS, which results in a form of MEP suppression named short-latency afferent inhibition (SAI) (30, 31). The same principle has been applied to investigate connectivity between M1 and visual (32, 33) or nociceptive (34) afferents.

TMS also has the ability to induce plastic changes to specific cortical sites and connected areas *via* rTMS protocols, which employ regular or patterned trains of TMS pulses to induce changes in cortical excitability that outlast the period of stimulation. These aftereffects are dependent on the intensity and frequency of stimulation; for example, rTMS applied at <1 Hz induces MEP suppression, whereas stimulation at >1 Hz induces MEP enhancement (35). Theta burst stimulation (TBS) is a variant of rTMS that induces plasticity by delivering bursts of stimulation (36–38). If bursts are separated by pauses, the protocol is termed intermittent TBS (iTBS) and is generally though to increase cortical excitability (39, 40); by contrast, in continuous TBS (cTBS), bursts are delivered without pauses, and the result is usually a decrease in cortical excitability (41–43). Paired associative stimulation (PAS) is another plasticity protocol that repeatedly pairs peripheral nerve stimulation with TMS over the contralateral M1 (44, 45), and is thought to operate through spike-timing dependent plasticity mechanisms (46) mediated by NMDA receptors (47, 48).

## Motor Neuron Disease

Motor neuron disease (MND) is a fatal, neurodegenerative disease, which has no effective cure or treatment. It is characterized by progressive degeneration of upper (UMN) and lower motor neurons (LMN) that make up the CST and results in profound weakness of affected muscles. Different clinical subtypes of MND are derived from the differential involvement of UMNs and LMNs, such that amyotrophic lateral sclerosis (ALS) describes UMN and LMN degeneration, primary lateral sclerosis (PLS) refers to a pure UMN syndrome and primary muscular atrophy (PMA) and spinal-bulbar muscular atrophy (SBMA/SMA) are characterized by pure LMN damage. Bulbar and pseudobulbar palsy affect LMN and UMNs of the bulbar muscles, respectively.

The diagnosis of MND is one of exclusion and is made using a combination of history, clinical examination, and neurophysiological testing. The latter mostly involves needle EMG, which is able to assess degeneration of LMNs. However, EMG findings may not be entirely specific, due to the existence of disorders which can mimic MND in terms of damage to LMNs. Conversely, an electrophysiological marker of UMN function should differentiate MND from mimic disorders, as UMNs are affected in MND but not in mimics. TMS has been proposed to serve as a tool to assess UMN function in MND, as detailed in the following sections.

### Corticospinal Excitability Measured in Motor Neuron Disease

As ALS is the most common form of MND, this is the most commonly examined model. From the perspective of spTMS measures, the RMT is lower in patients with MND than age-matched healthy controls. However, some studies have found no difference in motor threshold between patients and healthy, age-matched controls (49–52). Given the differential involvement of UMNs and LMNs in ALS, and that degeneration continues as disease progresses, the discrepancies in RMT may arise from differences in disease duration and UMN/LMN susceptibility. Indeed, the RMT is higher in patients with PLS than those with ALS and healthy controls (53) and raised in patients with mixed UMN and LMN signs (51). Conversely, the RMT is unchanged in patients with SMA, a LMN variant of MND (54). This bias considered, TMS studies seem to suggest corticospinal hyperexcitability in MND. However, patients with MND are excluded from TMS testing if MEPs cannot be generated, which is attributed to an unexcitable cortex. Calanie et al. have suggested that removal of this group of patients constitutes a form of selection bias, whereby individual patients with raised RMTs are excluded, thereby decreasing the group-level RMT (55).

The nature of this cortical hyperexcitability has been further investigated with ppTMS. A reliable finding from ppTMS studies has been an impairment of SICI in ALS (56–58), pointing to a breakdown of inhibitory transmission involving GABA_A_ receptors. Other forms of cortical inhibition seem to be lacking as well in ALS, as suggested by shortening of CSP, which is commonly associated with GABA_B_ receptors activity (49, 59, 60), and by reduction of SAI (61). ppTMS measures of intracortical facilitation in ALS are less clear, with some studies showing increases (58, 62–64) and others showing no change (50, 56, 65), compared to healthy controls.

### TMS Informs Clinical Features and Therapies in Motor Neuron Disease

The split-hand phenomenon, typically found in ALS, describes the selective wasting of the thenar eminence of the hand over the hypothenar eminence (66). This could be explained by differential sensitivity of LMNs or UMNs to neurodegeneration. Indeed, the cortical representation of the abductor pollicis brevis (APB), a muscle of the thenar eminence, shows more excitability (measured as MEP/CMAP ratio) and less SICI compared to that of abductor digiti minimi, a muscle of the hypothenar eminence (67, 68). Whilst both the APB and flexor pollicis longus (FPL) muscles are supplied by the C8 and T1 roots *via* the median nerve, wasting preferentially affects the APB. This is corroborated by greater cortical innervation of the APB muscle than FPL (69, 70). Hence controlling for the peripheral component of the MEP by expressing the MEP as a proportion of the CMAP, and measuring MEPs from muscles supplied by the same roots and nerves suggests a cortical origin of the split-hand phenomenon.

Fasciculations are another key feature of MNDs and are thought to reflect muscle denervation resulting from LMN degeneration (71); as such, they are found is a large number of diseases involving the spinal cord. MND represents a special circumstance, since fasciculations occur in the presence of concurrent UMN and LMN degeneration. The origin of fasciculations in MND has been investigated by assessing central and peripheral nerve excitabilities using TMS. A significant, negative correlation between corticospinal inhibition (measured *via* the GABA_B_-mediated CSP) and the frequency of fasciculations has been found in ALS but not in disorders characterized by peripheral nerve hyperexcitability (cramp-fasciculation syndrome, benign fasciculation syndrome, Morvan's syndrome and Isaac's syndrome), suggesting that fasciculations in ALS have a cortical contribution (72). The finding that cortical dysfunction contributes to fasciculations speaks to the top-down hypothesis of MND, where degeneration starts in the UMN and progresses along the motor neuroaxis.

Much attention in TMS-MND literature has been paid to the investigation of hand muscles, probably due to their large cortical representation. However, MND also affects other muscles, which are clinically relevant; degeneration of bulbar muscles can result in difficulties in swallowing, which confers a poorer prognosis (73). The involvement of bulbar muscles in MND has been assessed using TMS (74–77). In some studies, subclinical involvement of bulbar muscles measured by TMS has been observed, although the lack of longitudinal assessments makes the clinical relevance of these findings uncertain. One barrier to this approach is that some patients cannot tolerate intra-oral recording electrodes (78). Alternative strategies using less invasive approaches such as ultrasound may circumvent the difficulties posed by intra-oral electrodes (79).

Riluzole is the only disease-modifying drug that exists for MND (80). How Riluzole exerts its beneficial effect is currently unknown, although TMS studies have given some insights. TMS studies have confirmed that Riluzole exerts an anti-glutamatergic effect by showing that its administration blocks NMDA-dependent plasticity induced by PAS (81). In addition, the administration of Riluzole restores impaired SICI in ALS (65, 82, 83) and restores peripheral nerve excitability (assessed using peripheral nerve stimulation) (84).

### Distinguishing Different Types of Motor Neuron Disease

TMS findings in other forms of MND are in keeping with the site of primary pathology; MEP amplitudes and CSP are both decreased in PLS than healthy controls, suggesting impaired corticospinal inhibition (77, 85). The RMT has been found to be higher in PLS than ALS (53, 86). In the study by Agarwal et al., patients with PLS had slower rates of disease progression than those with ALS; it may be the case that the lower RMT in ALS is a pathological manifestation of hyperexcitability, not as prominent in PLS, and hence suggests differential involvement of corticomotor neurons in PLS. On the other hand, the lower RMT in ALS may be due to LMN degeneration in ALS. The inability to differentiate these competing hypotheses highlights the limitation of the MEP as a readout of M1 function.

TMS studies in LMN diseases such as SMA and monomelic amyotrophy (MMA) are generally in keeping with the notion of UMN preservation (54, 87–90). Specifically, the study by Farrar et al. has shown that measures of peripheral nerve excitability (CMAP amplitude, F-wave latency and frequency of F-waves occurrence), SICI and the MEP:M-wave ratio in patients with SMA is comparable to that found in healthy controls. Furthermore, SICI is greater in patients with SMA than those with ALS (88). These findings of retained corticomotoneuronal function are consistent in patients with MMA (90) and show that corticomotoneuronal dysfunction does not drive the LMN dysfunction in SMA and MMA. These studies indicate how TMS can provide insights into different motor neuron diseases by showing differential involvement of M1.

Given that genetic causes of MND exist, carriers of mutations in involved genes provide an interesting model to study presymptomatic MND, since the temporal consequences of neurodegeneration in MND can be explored as the disease manifests and develops. Corticospinal hyperexcitability, measured as reduced SICI and greater ICF, precedes the development of symptoms in presymptomatic SOD-1 (91) and *C9orf72* (92, 93) carriers, suggesting that hyperexcitability represents a hallmark of degeneration. However, in other studies, SOD-1 mutation carriers have shown no difference in CSE measures compared to healthy controls (94). In a study by Turner et al., corticospinal hyperexcitability was found in patients with sporadic ALS but not SOD-1 ALS (95), suggesting that, despite similar clinical presentations in sporadic and familial ALS, the mechanisms by which neuronal degeneration occurs may differ.

### Distinguishing Motor Neuron Disease From Mimic Disorders

The findings offered by TMS have culminated in the ability to use neurophysiological indices to differentiate MND from mimic disorders, under the assumption that UMNs are affected in the former but not the latter. SICI, ICF, and CSP have all been shown to differentiate ALS from mimics (such as multifocal motor neuropathy, chronic inflammatory demyelinating polyneuropathy, and distal hereditary motor neuronopathy) with receiving operator characteristics (ROC) area under the curve (AUC) of 0.76 for SICI (96). In a prospective, multicentre study by Menon et al., TMS was able to differentiate MND from mimic disorders with a sensitivity of 73.21% and a specificity of 80.88% (97). Consistent with previous literature, SICI was found to be the major neurophysiological parameter differentiating MND from mimics. The authors estimated that the diagnosis of MND could be improved by up to 15.8 months, a timeframe considered to be clinically significant, given the potentially short life expectancy. One flaw of this study is that TMS is unlikely to be used in isolation in the diagnosis of MND. The same group of investigators have developed a diagnostic index for ALS (ALSDI), which incorporates age, anatomical site of disease, CSP duration, CMAP amplitude and SICI to differentiate ALS from mimic disorders (98). The ALSDI was able to differentiate ALS from mimic disorders with 83.3% sensitivity, 84% specificity, and 83.5% diagnostic accuracy, which constitutes a mild increase relative to TMS alone (97). Of note, of 133 patients originally classed Awaji negative, 38 were reclassified as having ALS using the ALSDI criteria, of whom 31 went on to develop ALS.

These studies show that TMS may have a diagnostic role in MND, but there are some limitations to its clinical use. Many of the studies outlined thus far have excluded patients if MEPs could not be generated. For example, in the study by Menon et al., patients were excluded if the maximum MEP generated was <1 mV in amplitude (97). Under this criterion, and given the muscle wasting found in MND, a significant proportion of patients may not be eligible for assessment. As MND is a degenerative disease, it may be the case that the utility of TMS to inform diagnosis changes as the disease progresses (99). Hence it is likely that TMS may have a specific role to play at a certain point in the diagnostic pathway, for example clarifying UMN dysfunction when clinical examination is ambiguous.

### Lessons From Motor Cortical Stimulation in Motor Neuron Disease

We have shown how assessing the function of the CST and M1 intracortical circuitry by means of TMS can give insight into MND pathophysiology. Corticospinal hyperexcitability, driven by a loss of intracortical inhibition, predominates as a key feature of MND pathophysiology. Extensive characterization of corticospinal changes in MND has resulted in a better understanding of clinical features, such as the split-hand phenomenon, and has promoted TMS as a diagnostic and prognostic tool for MND. Viewing TMS through the lens of MND has also highlighted some of its key limitations. Most notable is that the MEP is not a pure measure of UMN function and relies on the LMN system for its generation. Consequently, patients are routinely excluded from investigation if MEPs of a satisfactory amplitude cannot be generated. This means that TMS use is largely confined to early and mid-stage MND, when muscle wasting is limited.

## Dementias

Dementia is an umbrella term for progressive disorders characterized by the loss of cognitive abilities such as memory, problem-solving, visuospatial awareness, behavior and language. Alzheimer's disease (AD), the most common cause of dementia, includes ~60–80% of all dementias and is characterized mainly by memory impairment and loss of visuospatial awareness. Notable features of dementia with Lewy bodies (DLB), the third most common type of dementia after AD and vascular dementia, include impairment of executive function, fluctuating cognition, visual hallucinations, and parkinsonism. Another common form of dementia is Frontotemporal dementia (FTD), that describes degeneration of the frontal and temporal lobes and presents with impairment in decision-making and behavioral control, personality changes, and language decline. Interestingly, FTD has a genetic and pathological overlap with MND (100, 101). In this review, we use these three conditions as an example to show how TMS can inform about diseases that do not primarily affect M1.

### Corticospinal Excitability in Dementias

Traditionally, TMS has been limited to cortical areas where an output can be easily measured; these include M1 and the visual cortex. Consequently, much of the early TMS literature focussed on M1 stimulation to infer cortical dynamics of diseases that do not primarily affect the motor system, as in dementias.

Most studies report an increase in CSE in AD, but decreased (102) or normal (103, 104) CSE have also been found. Investigations with ppTMS have shown reduced intracortical inhibition but normal intracortical facilitation (103, 105–107). The clinical relevance of corticospinal hyperexcitability is currently unknown. It may be possible that it represents a subclinical feature of M1 dysfunction. For instance, AD patients exhibit subtle locomotor and parkinsonian signs on examination (108), and cortical myoclonus, that is related to M1 hyperexcitability, is a common late feature of AD (109).

Cholinergic dysfunction is a hallmark of AD and implicated in its typical cognitive impairment (110). SAI pairs a peripheral sensory stimulus with a TMS pulse applied to the contralateral M1, which results in a decrement of the MEP compared to unconditioned TMS. Pharmacological manipulation has shown that SAI is sensitive to cholinergic neurotransmission (111) and as expected, SAI is reduced in AD (104, 106, 112–114). Moreover, anti-cholinesterase medications, commonly used in the management of AD, restore impaired SAI (112, 115, 116), although not all studies have confirmed this result (31, 117). Baseline SAI and increase in SAI after one dose of rivastigmine correlated with response to long-term treatment. Conversely, no SAI response to rivastigmine was associated with poor long-term clinical response to rivastigmine, thereby raising the possibility that SAI might predict the efficacy of future anti-cholinesterase treatment (116). Interestingly, anti-cholinesterase therapy also reverses impaired intracortical inhibition in AD (103, 105), suggesting that cholinesterase inhibitors exert effects on non-cholinergic circuits.

Only one study has investigated interneuronal circuits in DLB, showing that SICI and SAI are not statistically different from healthy controls (106). Early studies of CSE in patients with FTD showed no changes compared to healthy subjects (103, 118). However, a series of studies by Benussi et al. have investigated intracortical circuits in a large number of FTD patients, finding that both intracortical facilitation (ICF and SICF) and inhibition (SICI and LICI) are lower in FTD than in healthy controls (119–121).

### Plasticity Protocols in Dementias—Going Beyond Corticospinal Excitability

rTMS protocols have allowed the study of synaptic dysfunction and impaired mechanisms of cortical plasticity in dementias, which are distinct from the changes in CSE. The aftereffects induced by rTMS applied over M1 are of smaller magnitude in patients with AD compared to healthy, age-matched control subjects, regardless of the protocols used and the direction of the effect; this finding is borne out over a variety of TMS plasticity protocols such as 1 and 5 Hz rTMS (122, 123), iTBS (124), and PAS (114, 125).

Smaller effects of several plasticity-inducing rTMS protocols suggest a global impairment in cortical plasticity that spans various different mechanisms. However, a study using variants of TBS has highlighted that specific types of plasticity may be preferentially affected in AD. Koch and coworkers found that the response to iTBS was significantly impaired in patients with AD relative to age-matched healthy controls. Conversely, the response to cTBS was not statistically different between groups. To explain their findings, the authors cited *ex vivo* work, where beta-amyloid oligomers obtained from cerebrospinal fluid (CSF) from patients with AD were shown to impair long-term potentiation (LTP) in rat CA3-CA1 synapses (126). In contrast, administration of beta-amyloid oligomers to the CA1 region in mice enhanced long-term depression (LTD) (127). The authors therefore posed that the impaired LTP-like plastic effects seen in humans was consistent with the effects of beta-amyloid pathology found in AD (124).

Whilst using evidence from animal and *ex vivo* research is useful in contextualizing TMS findings in patients, their inferences are weakened given that these mechanisms may not be applicable *in vivo*. Indeed, the terms LTP-like and LTD-like (rather than LTP and LTD) are used to describe changes in CSE observed in humans after plasticity protocols; this nomenclature is used due to the uncertainty that what is observed in humans is equivalent to what is observed *in vitro*. Therefore, concurrent measures of pathological features of disease, using fluid biomarkers, should be combined with TMS protocols to help bridge the gap between neuropathology and network neuroscience. By measuring CSF total Tau (t-Tau), phosphorylated Tau (p-Tau), and amyloid beta 42 (Aβ42) in AD patients undergoing 1 Hz M1 rTMS, Koch et al. have found that rTMS-induced inhibition correlated with t-Tau but not Aβ42 (123). By measuring the extent of amyloid and Tau pathology *in vivo*, the authors have identified a potential pathological substrate responsible for impaired plasticity in AD.

### TMS Measures Correlate With Cognitive Symptoms and Predicts Functional Decline

It has been shown that some TMS measures of motor function correlate with clinical features in dementias. For instance, CSE, as measured by the RMT, correlates with cognitive severity in AD assessed by the mini mental state examination (MMSE) (105, 128–130). Furthermore, impairment in SAI is related to parkinsonian motor signs and CSF Aβ_42_ levels (108). The increase in CSE with disease progression may be a consequence of accumulated pathology; indeed, neurofibrillary tangles, and amyloid plaques are also found in M1 (131).

The relevance of particular neurophysiological markers may differ among different types of dementia. For example, whilst SAI is reduced in both AD and DLB, it is correlated with behavioral disinhibition in AD and hallucinations in DLB (113), probably due to the diverse actions of acetylcholine. On the other hand, different symptoms within the same disorder may be related to different interneuronal circuits; Benussi et al. have found, in a large sample of patients with FTD, that negative symptoms (apathy and indifference) are correlated with ICF, whereas positive symptoms (irritability, impulsivity, and aggressiveness) are correlated with SICI (121).

Longitudinal assessments in patients with neurodegenerative diseases allow investigators to examine which markers may be predictive of clinical and functional decline. In a comprehensive study by Koch et al., CSF Tau, LTP-like plasticity (measured using iTBS) and APOE status (APOE polymorphic alleles are the main genetic determinants of AD) were assessed in patients with AD. They found that the effect of iTBS was reversed in APOE3 patients, resulting in a decrease in post-protocol CSE, while APOE4 patients, that are at increased risk of AD compared with those carrying the more common E3 allele, did not show any significant changes in CSE after iTBS. In addition, higher CSF tau levels (an AD biomarker) were associated with a greater impairment of LTP-like plasticity and faster disease progression in patients with AD (132). The response to iTBS has also shown to predict disease progression in AD such that the greater the increase in CSE after iTBS, the lower the probability of disease progression (133).

SICI, SICF, and LICI, are all reduced in FTD. SAI is not different in patients with FTD compared to healthy subjects; this is to be expected given that SAI reflects cholinergic transmission, which is not typically involved in FTD. As well as being correlated with the FTLD-CDR (a clinical rating scale to measure FTD severity), it was found that less SICI is associated with greater clinical progression of FTD (120). The interpretation was that, rather than reflecting impairments of a specific interneuron circuit, these abnormalities reflect an inability to integrate two, closely timed pulses. As seen above in MND, patients with a genetic susceptibility to disease provide an insight into prodromal stages. To that end, SICI, ICF, and LICI have been shown to be altered before clinical onset in carriers of *GRN* and *C9orf72* mutations. By calculating the estimated time from symptom onset, it was found that TMS measures can be altered up to three decades before the disease become manifest (119).

### Measuring Activity Outside of the Motor Cortex

A limitation of the aforementioned studies is that they give information about motor intracortical dynamics, despite the primary site of pathology lying outside M1. Previous findings may therefore not be indicative of cortical function outside M1, and hence erroneous conclusions may be drawn about cortical function in dementias. To address this, investigators have combined TMS with other imaging modalities, which reflect cortical excitability outside of M1. For example, concurrent TMS-EEG allows measurement from virtually all areas in the brain convexity. TMS pulses result in EEG perturbations, which can be measured in the time domain, as TMS-evoked potentials (TEPs), or in the time-frequency domain, as TMS-induced oscillations (134, 135). This novel approach has circumnavigated many of the traditional barriers to applying TMS outside of M1, and also allows to greatly expand the number of measured variables relative to TMS alone. TMS-EEG has been applied to patients with AD and mild cognitive impairment (MCI), finding a reduced P30 component in temporo-parietal regions, consistent with the site of primary pathology (136). Although in a small sample, the amplitude of the P30 component was able to differentiate patients with AD and MCI with an AUC > 0.90. In addition, a smaller P30 component was associated with a greater clinical dementia rating score (137). spTMS to the left superior frontal cortex (Brodmann areas 6 and 8) found that excitability (TEP amplitude and significant current density) was reduced in patients with AD relative to healthy, age-matched controls. As this study measured TEPs in young and old healthy controls, Casarotto et al. surmised that TEPs were not abnormal unless affected by some pathological process, such as amyloid/tau deposition in AD (138). Although previous studies have found cortical excitability to be decreased in AD relative to healthy controls, cortical excitability (measured as the global mean field power—GMFP) was found to be higher in AD by Ferreri et al. (139). Further, better powered studies are required to clarify how cortical excitability, as measured by TMS-EEG, is affected in AD. In particular, one caveat of TEPs, including the P30 component, is that their amplitude can be spuriously decreased by the increased scalp-to-cortex distance in patients with cerebral atrophy. This factor should be accounted for in future studies using TMS-EEG in dementia.

TMS has seldomly been used to investigate functional connectivity in AD. By a twin-coil approach, altered parieto-motor connectivity has been found in AD, and this impairment is positively correlated with derangement in episodic memory and executive function (117). However, as mentioned twin coil approaches are limited to assessing connectivity between a ROI and M1, so they might not be necessarily informative of degeneration outside M1.

### Differentiating Types of Dementia

As with MND, attempts have been made to stratify and diagnose dementias based on neurophysiological parameters. Neurophysiological assessment in patients with MCI with and without features of AD has found that SAI is lower in latter group. When incorporating other measures of intracortical function such as SICI and SICF, these neurophysiological parameters were able to differentiate the two with a sensitivity of 94.4% and specificity of 87.9% (140). The same approach has been used to differentiate three types of dementia: AD, DLB, and FTD. In this large study, a combination of TMS measures, including SICI, ICF, SAI, and LICI were incorporated into a random forest classifier, which was able to cluster dementias with high precision (0.86–0.92) and recall (0.93–0.98). Binary classification accuracy ranged from 0.89 to 0.92, showing that TMS measures can help diagnose specific dementias with high accuracy (141).

Although these results are encouraging, it is unlikely that TMS alone will be used to diagnose specific dementias. It is therefore important to assess how TMS can change diagnostic certainty when approaching dementias. This has been assessed in the diagnosis of AD and FTD, whereby diagnostic certainty from three approaches was compared: clinical workup alone (demographic, clinical and neuropsychological evaluation), clinical workup + amyloid markers (CSF or amyloid positron emission tomographic imaging), and clinical workup + TMS intracortical connectivity measures. TMS measures were found to increase the discrimination between AD and FTD compared to clinical evaluation alone. The classification accuracy using clinical workup + TMS measures (AUC = 0.98) was similar to that of clinical workup + amyloid markers (AUC = 0.99) (142). This same approach has been used to differentiate MCI-AD, MCI-FTD, MCI-DLB, and MCI-other. The findings in this study replicate those in the previous study, that TMS measures increase the diagnostic certainty of MCI variants and that the increase is comparable to CSF fluid biomarkers (143). The non-invasive nature of TMS may be an advantage over fluid biomarkers, given that the addition of TMS measures or CSF biomarkers are comparable in diagnostic accuracy.

Some limitations of these studies should be mentioned. Firstly, they are retrospective, and neurophysiological parameters are fitted to explain known diagnoses. This commonly leads to overfitting of the data, which results in poor predictive performance in a prospective cohort. Secondly, according to clinico-pathological studies, specificity of clinical diagnosis of neurodegenerative disease, and especially tauopathies (including non-primary tauopathies such as AD), is low (144). Therefore, the reliability of TMS as diagnostic tool based on clinical diagnosis should be considered with caution in this context. An appropriate test for diagnostic utility of TMS in dementias would therefore be to perform a prospective study in a clinical setting, with comparisons of diagnostic accuracy with and without TMS.

### Lessons From TMS in Dementias

TMS studies have established corticospinal dysfunction in an array of dementias that track the severity, symptomatology, and progression of disease. The addition of TMS measures to clinical assessment may enable an increase in diagnostic accuracy for dementia variants and shows potential for appropriate clinical translation, although appropriate prospective trials are required. One key caveat of TMS studies in dementia is that they usually study M1 function; the findings in M1 may not generalize to other cortical areas where pathology may be more clinically relevant. However, this view might be challenged by recent findings of widespread synaptic loss in AD, including pericentral regions, assessed with SV2A PET (145). Combination with other imaging modalities, such as EEG, has enabled the wider effects of TMS to be evaluated, allowing for cortical function of clinically significant areas to be assessed. Whilst yielding valuable insights into cortical excitability and functional connectivity outside of M1, only a small number of TMS-EEG studies have been performed in dementia. Future experiments should aim to further establish the wider effects of TMS and investigate the role of non-motor regions in dementias.

## Parkinsonisms

The term parkinsonism describes a collection of disorders characterized by the presence of bradykinesia in combination with either rest tremor, rigidity or both. The most common form of parkinsonism is Parkinson's disease (PD), an idiopathic neurodegenerative disease neuropathologically distinguished by Lewy bodies and Lewy neurites, which are neuronal inclusions immunopositive for the protein α-synuclein (146). Other neurodegenerative parkinsonian syndromes include multiple system atrophy (MSA), corticobasal syndrome (CBS), DLB and progressive supranuclear palsy (PSP). The common feature in these conditions is that they present with the cardinal parkinsonian features plus atypical signs, however they differ in the primary protein implicated in neurodegeneration or in the distribution of cortical proteins. For example, although PD, MSA, and DLB are α-synucleinopathies, their pathology is significantly different, including the cell type involved (neurons in PD and DLB and oligodendroglia in MSA) and the degree of neuronal loss (only in selected regions in PD but widespread throughout many regions in MSA) (147). On the other side, both PSP and CBS have neuronal and glial lesions that are composed primarily of hyper phosphorylated tau (148, 149). However, the common denominator of all degenerative parkinsonisms is loss of dopaminergic neurons of the substantia nigra pars compacta. This section will discuss how TMS can inform about parkinsonian syndromes that primarily affect subcortical structures without a prominent involvement of higher-order brain areas.

### Corticospinal Excitability in Parkinsonisms

The majority of TMS literature in parkinsonisms has been performed in PD, which generally present with increased CSE compared to healthy controls (150–152). Dissecting this increase in CSE with ppTMS has resulted in conflicting results, with some groups reporting normal intracortical inhibition and facilitation (153–156), whereas others describe abnormalities in these measures (155, 157–160). Sensorimotor interaction, as assessed with SAI, has also shown mixed results (161–166). The conflicting outcomes may be partially explained by clinical features of PD, which could be variable among patients. Inhibitory deficits have been found in early, untreated PD (167) and CSE hyperexcitability is greater in the more clinically affected hemisphere (159, 168). Failing to account for disease duration or clinical severity may explain the discrepancies in intracortical inhibition and facilitation; indeed, TMS measures vary as a function of symptomatology (see below).

As aforementioned, there are other neurodegenerative causes of parkinsonism that have been investigated using TMS. In a comparison with patients with PD, those with CBS and PSP have reduced SICI and raised SICF (118, 169, 170). This is consistent with the cortical Tau pathology found in CBS and may therefore provide an objective marker of cortical involvement. Conte et al. have found a specific aberration of SICI but not ICF in patients with PSP, suggesting a specific impairment of inhibitory interneurons (171). Another example of differential cortical involvement comes from the comparison between Richardson syndrome and PSP-Parkinsonism; transcallosal inhibition is affected more in Richardson syndrome than PSP-Parkinsonism and PD (172), which may be due to higher cortical tau burden in Richardson's syndrome than PSP-Parkinsonims and PD. Furthermore, SAI has been found to differ between patients with PSP and those with PD, suggesting differential cholinergic involvement and sensorimotor interaction between these two (162, 170). Initial studies in MSA showed no sensory or motor threshold abnormalities, but did find prolonged CMCT in the lower limbs (173, 174), potentially owing to CST involvement seen in MSA. Sensorimotor processing and cholinergic function, as indexed by SAI, has been found to be significantly impaired in MSA compared to PD and correlates with clinical neuropsychiatric measures (175, 176).

Parkinsonisms due to an identifiable genetic cause differ from PD, both pathologically and clinically (177); this difference extends to electrophysiological assessments with TMS. CMCT is raised in patients with *Parkin* mutations (154, 178), implying subtle corticospinal dysfunction not found in PD. Patients with *LRRK2* gene mutations exhibit less SICI and more ICF than patients with PD (179, 180). I/O curves and the CSP of patients with *SNCA* mutations differ from tremor-dominant PD but not akinetic PD (181). As with MND and dementias, genetic models of disease provide key insights into prodromal and preclinical disease pathophysiology. Indeed, subclinical *Parkin* and *PINK1* mutation carriers display hyperexcitable premotor-M1 connectivity measured with twin-coil TMS (182).

Patients with PD also show impairment in various different types of plasticity. LTP-like plasticity, as measured by MEP enhancement after iTBS, is impaired in PD (183–187), irrespective of medication status (188) or levodopa-induced dyskinesias (LIDs) (189). Spike-time-dependent plasticity, measured using PAS, is also impaired compared to healthy controls (190). However, some studies show a preservation of plasticity in patients with PD (191, 192). As with measures of CSE, the varied findings concerning plasticity in PD may have arisen due to uncontrolled variables. For example, there is an association between dopaminergic medication and cortical plasticity (193–195), and the presence or absence of LIDs (196).

Parkinsonisms have been investigated with stimulation of brain areas other than M1. Cerebellar stimulation is usually delivered in the form of rTMS, which has downstream effect on CSE (197), or spTMS, which can coupled with M1 TMS to obtain cerebellar-brain inhibition (CBI) (198). CBI is a form of ppTMS in which a conditioning stimulus over the cerebellum is followed by a TMS stimulus at M1, with an ISI of ~5 ms. CBI is achieved as a decrement in the MEP amplitude. Both cerebellar plasticity (199) and CBI (200) have been found to be impaired in patients with PD, which has been interpreted as an impairment in cerebello-thalamo-cortical connectivity. In the study by Ni et al., postural tremor associated with PD was reset by cerebellar and M1 TMS, whereas rest tremor was reset by M1 TMS only, implying that the two types of tremor operate *via* different pathways (200). Schirinzi et al. found that CBI was impaired in patients if they had evidence of a dopaminergic deficit on imaging (187) suggesting that impairments in CBI may be a surrogate marker of dopamine deficiency.

### TMS Correlates of Symptomatology in Parkinsonisms

An effort has been made to investigate the clinical relevance of TMS measures in parkinsonisms to elucidate whether TMS abnormalities represent an epiphenomenon, rather than being related to any particular clinical feature or pathological process (201).

Bradykinesia is the cardinal feature of parkinsonism; it generally describes slowness of movements, which is reflected, for example, in the decrement in amplitude and velocity of repeated movements, or in increase reaction times (202–207). A steeper I/O curve is found in patients with PD, suggesting a greater gain of M1 than healthy controls (160). Furthermore, the slope of this curve correlates with clinical measures of bradykinesia. The authors proposed that, whilst counterintuitive, this increase in M1 gain may represent a compensatory mechanism to decreased excitatory input from the basal ganglia. Alternatively, the increase in M1 CSE could represent a form of noise, which competes with the motor instruction from the basal ganglia, thereby making movements more difficult to execute. If the latter hypothesis were true, then CSE should increase as the disease progresses (presumably as bradykinesia increases), but this has not been tested yet. A role of posterior parietal cortex (PPC)-M1 connectivity in bradykinesia has been proposed using a twin-coil TMS approach. PPC-M1 TMS at ISI 4 ms results in an increase in MEP amplitude in healthy controls, that is not found in patients with PD, irrespective of dopaminergic medication intake (208). By integrating this paradigm with behavior, the authors observed that greater strength of PPC-M1 connectivity was associated with faster movement execution in healthy controls, and PD patients on and off medication. The assessment of M1 function during movement therefore permits a contextually appropriate inference of the role of M1 CSE in bradykinesia.

A feature of parkinsonism with chronic levodopa treatment is the development of LIDs. LIDs are involuntary movements thought to arise as a consequence of nigrostriatal depletion and erratic cerebral levodopa concentrations. The presence of LIDs has generally been associated with M1 hyperexcitability, characterized by increased ICF and SICF (209–211) and decreased SICI and LICI (211, 212). LID generation appears to be linked to a wider dysfunction in the cortical motor network, as suggested by a weaker inhibitory functional connectivity between the inferior frontal cortex (IFC) and M1 (210). An interesting line of research has been devoted to clarifying the relationship between TMS measures and levodopa administration in patients with LIDs. Acute levodopa administration leads to an abnormal increase in excitability in the supplementary motor area (SMA) in patients with LIDs; this occurs in the cortex contralateral to the most affected body side, in comparison with the less affected hemisphere (213) and with PD patients without LIDs (214). It is well-known that LIDs are associated with chronic levodopa exposure. However, the latter might be necessary but not sufficient to cause LIDs (215), and it is not entirely clear to what extent degeneration linked to PD itself might be implicated. An indication on the role of levodopa on LED development comes from the study of Zittel et al. (216), who found that first-time exposure to levodopa exerts different effects on PMd-M1 inhibitory connectivity than chronic dopaminergic stimulation in PD, suggesting a change in the responsiveness of cortico-cortical circuits during the course of disease. At least part of this change in responsiveness might be due to deranged control of cortical plasticity, as suggested by Morgante et al. (217). These authors found that LTP-like plasticity induced by PAS is restored by levodopa administration in non-dyskinetic, but not in dyskinetic PD patients. This suggests that abnormal synaptic plasticity in the M1 may play a role in the development of LIDs, a notion which has been confirmed by a number of other groups (188, 196).

### TMS Informs Therapies Used to Treat Parkinsonisms

Dopaminergic replacement therapy is the mainstay of treatment for parkinsonisms, albeit mostly beneficial in PD compared to the other forms. As well as ameliorating parkinsonian motor features, it also results in restoration of aberrant neurophysiological measures such as SICI, LICI, and the CSP (152, 160, 194, 218, 219). However, the correction of these abnormalities does not necessarily imply that specific inhibitory or excitatory circuits mediate the effects of dopaminergic therapy; rather, they could be in keeping with the restoration of normal movement.

Deep brain stimulation (DBS) is a surgical treatment used for advanced stage PD, which involves the implantation of electrodes in order to stimulate subcortical nuclei of the basal ganglia. Common targets include the subthalamic nucleus (STN) and globus pallidus pars interna (GPi). Although the precise mechanism by which DBS exerts its therapeutic effect is not entirely known, there is evidence from functional neuroimaging that DBS normalizes activity and connectivity in the basal ganglia motor circuit (220, 221). This is consistent with TMS findings that STN (222–228) and GPi (224) DBS normalize aberrant M1 intracortical facilitation and inhibition.

Historically, functional neurosurgery involved lesioning of subcortical nuclei, which resulted in clinical benefit of motor symptoms. An important distinction is the difference between DBS and subthalamotomy/pallidotomy; the latter achieves clinical benefit by lesioning the STN or GPi, respectively. Despite targeting the same region, it is unknown whether the two methods achieve clinical benefit in the same way. Whilst neurophysiological measures are restored during DBS, they are not after pallidotomy (150). Hence, despite similar clinical outcome, DBS and lesioning approaches seem to have differing mechanisms of action. This suggests that stimulation in DBS actively alters cortico-basal ganglia circuits, a feature not found during OFF DBS conditions and lesioning. Additionally, cognitive side effects are common in DBS (229) whereas they are not in subthalamotomies (230). As shown by TMS studies, DBS has active effects on cortical functioning; this same phenomenon may be mediating the cognitive side effects during DBS.

Dopaminergic therapy and DBS exert similar clinical benefit for patients with PD, although there exist some notable differences in how these benefits arise. Similar improvements in SICI and CSP during STN DBS, apomorphine and levodopa are observed, suggesting that they share some common mechanisms in clinical improvement (224, 228). On the other hand, concurrent TMS-EEG shows that cortical excitability, as measured by the GMFP from 45 to 80 ms and high-alpha oscillatory activity are increased during STN DBS; conversely, GMFP from 80 to 130 ms and beta oscillatory activity are increased during DBS and levodopa (231). However, the clinical relevance of this finding is unclear, given that no correlation with clinical symptoms was performed in the study. It may be the case that the change in oscillatory activity and cortical excitability represents an epiphenomenon of levodopa administration or STN DBS, not related to clinical improvement.

The studies mentioned thus far have suggested that therapies to treat parkinsonisms act *via* cortical mechanisms. Whilst apomorphine restores SICI and CSP, it has no effect on Hoffman's reflex—a measure of spinal motor neuron excitability (218). On the other hand, STN DBS alters spinal excitability by restoring TMS-induced facilitation of Hoffman's reflex, showing that STN stimulation restores facilitatory drive to the spinal motoneuron pool (227).

### Lessons From TMS in Parkinsonisms

TMS in parkinsonisms informs about the role of one node (M1) in the cortico-basal ganglia-cortical network. Hence, findings from these experiments infer the effect of basal ganglia pathology on overall motor output. Contrary to MNDs, isolated measures of cortical function are less useful in informing disease pathophysiology of parkinsonisms. Instead, TMS is better suited when a specific question or aspect of disease is being investigated. For example, TMS has enabled subtle differences in therapies for parkinsonisms to be appreciated and has given insights into particular symptoms, such as LIDs and bradykinesia. Going forward, TMS may be useful to inform how novel therapies for parkinsonism, such as focused ultrasound therapy, act to exert clinical benefit.

## Recommendations for the Use of TMS in Neurodegenerative Diseases

**Figure 1:**
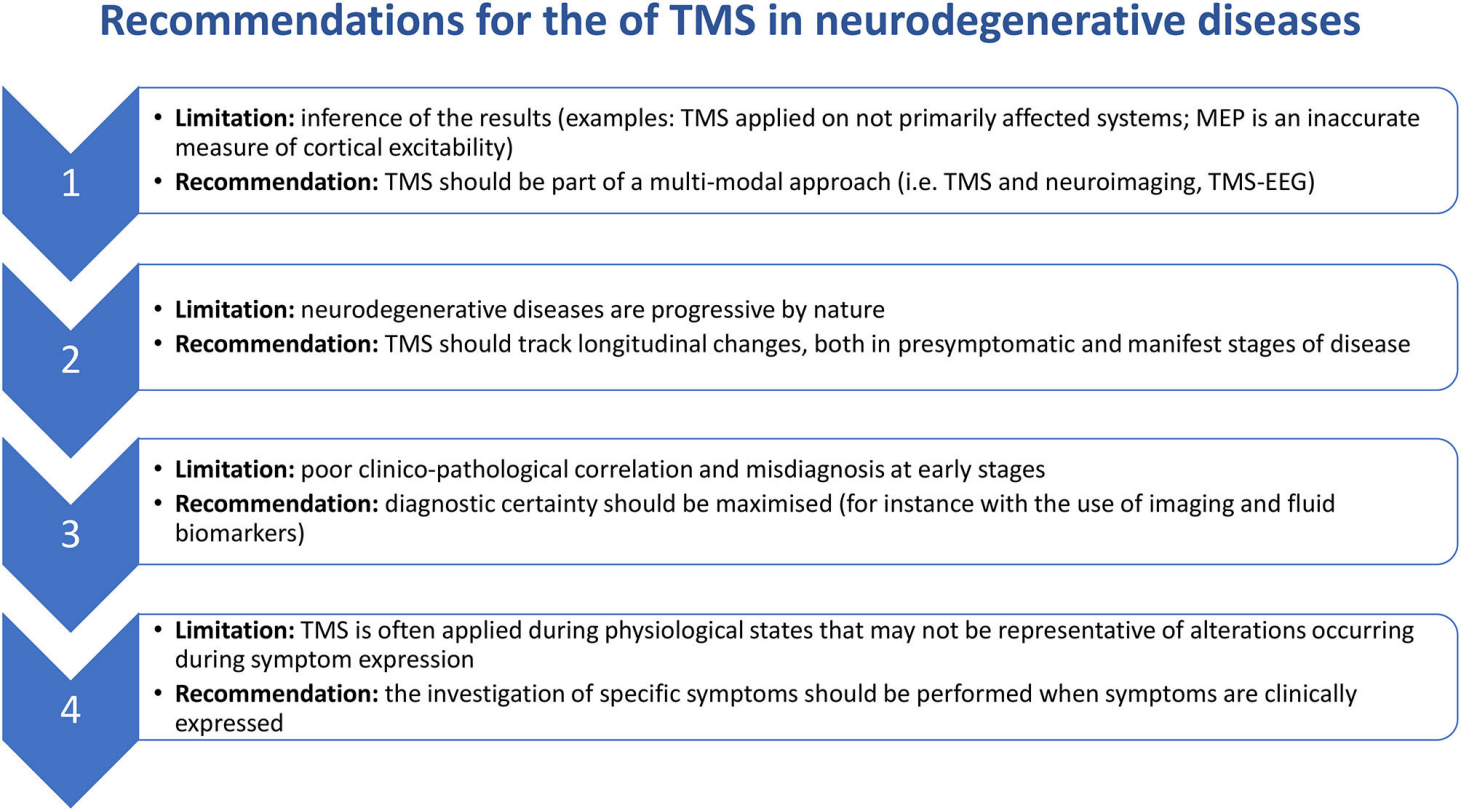
Recommendations for the use of TMS in neurodegenerative diseases.

This review has covered how TMS has been used to give insights into the pathophysiology of neurodegenerative diseases affecting cortical and subcortical structures (summarized in Table 1). Below, we synthesize some of the salient points gleaned in this review for researchers wishing to investigate neurodegenerative diseases in the future (Figure 1).

**Table 1:** Summary of corticospinal changes stratified by neurodegenerative disease.

	**RMT**	**SICI**	**LICI**	**CSP (length)**	**SICF**	**ICF**	**SAI**
**MND**							
ALS	Reduced	Reduced	-	Reduced	Raised/normal	Raised/normal	Reduced
PLS	Raised	Normal	-	Reduced	-	-	-
SMA/SBMA	Normal	Normal	-	-	Normal	Normal	-
**Dementias**							
AD	Reduced	Reduced	Reduced	Increased/normal	Normal	Normal	Reduced
DLB	Normal	Normal	Normal	-	-	Reduced	Reduced/normal
FTD	Normal	Reduced	Reduced	Normal	Reduced	Reduced	Normal
**Parkinsonisms**							
PD	Reduced/normal	Mixed	Mixed	Mixed	Mixed	Mixed	Mixed
CBS	Normal	Reduced	-	-	Raised	Reduced	Normal
PSP	Normal	Reduced	-	Reduced	Raised	Normal	Normal
MSA	Normal	Reduced	-	Raised	-	Normal	Reduced

*ALS, amyotrophic lateral sclerosis; PLS, primary lateral sclerosis; SMA/SBMA, spinal-bulbar muscular atrophy; AD, Alzheimer's disease; DLB, dementia with Lewy bodies; FTD, frontotemporal dementia; PD, Parkinson's disease; CBS, corticobasal syndrome; PSP, progressive supranuclear palsy; MSA, multiple system atrophy; “-”, not tested*.

*Neurophysiological measures for PD are mixed due to multiple confounding variables such as medication use, disease duration, and symptomatology. We refer the reader to the main test for elaboration*.

### Recommendation 1: TMS Should Form Part of a Multi-Modal Approach to Investigate Neurodegenerative Diseases

Traditionally, there has been no robust outcome measure for TMS outside of M1. Consequently, TMS experiments in disease states infer cortical function from M1-evoked MEPs, even in diseases where M1 is not primarily involved, such as dementias and parkinsonisms. Doing so might limit inferences about those TMS results to M1 only and away from sites of primary pathology. Additionally, any inferences made regarding M1 excitability may be confounded by several factors. One example is represented by inputs from other cortical areas, which are probably active during both resting conditions and active behavior (3, 11, 12). A second issue pertains to the influence of spinal cord circuitry on MEP (7), which is usually unmeasured and that can be a significant problem if cortical and spinal cord damage coexist. The limitations outlined above can be overcome by combining TMS with neuroimaging approaches to give a more comprehensive view of brain function. For example, the use of concurrent TMS and EEG has shown that temporo-parietal excitability is decreased in patients with AD compared to healthy controls (136), a finding that would not have been possible with M1 TMS alone. Due to its ability to measure cortical function without the confounds of peripheral sources, TMS-EEG may also allow to circumnavigate the caveats posed by MEPs in conditions of impaired neuromuscular function, such as MND (232).

### Recommendation 2: TMS Should Track Longitudinal Changes in Neurodegenerative Diseases

It is in the nature of neurodegenerative diseases to be progressive and evolve over time. As well as worsening of particular symptoms, new ones can develop, and diagnoses can even be revised. For example, patients can clinically resemble PD at onset, but progression might be compatible with other forms of parkinsonism. This raises a number of important points: firstly, the progression of symptoms and development of new ones allows longitudinal assessments to be of value in neurodegenerative diseases. Whilst longitudinal assessments in individuals would be ideal, the variability in TMS measures over time may be problematic (233, 234). An alternative might be to apply TMS to populations of patients to characterize disease progression, and then track how disease progression changes under different treatments. Longitudinal assessments will enable identification of which TMS measures scale with disease severity or symptomatology and which do not (235). It is also noteworthy that neurodegenerative disorders can have a long presymptomatic phase during which biomarkers can be positive; therefore, another possible application of TMS could be to track changes during this early stage.

### Recommendation 3: Diagnostic Certainty of Clinical Population Should Be Maximized

The third point concerns the diagnosis of patients enrolled into TMS studies. Pathological confirmation is considered the gold-standard of diagnosis but is not always viable. In most studies, the diagnosis of a particular neurodegenerative disorder is made upon clinical assessment; in some, two or more neurologists make an assessment to increase diagnostic certainty. However, clinico-pathological correlation is poor (236) and, as explained earlier, neurodegenerative diseases change over time, and a diagnosis at one point in time may be revised later on. These inaccurate diagnoses add noise to the patients' sample and hence the same pathological process might not be assessed in all patients. Longitudinal assessments are desirable, as they increase diagnostic accuracy. Furthermore, if a diagnosis is revised over time, then measures at the first time point may represent preclinical/pre-diagnostic markers. In all, multimodal approaches to confirm diagnosis should be encouraged. Additionally, validation of outcome measures, such as imaging (237) and fluid biomarkers (238, 239) developed to confirm diagnosis of neurodegenerative diseases, should be further prompted.

### Recommendation 4: TMS Investigating Symptomatology Should Be Applied During Symptom Expression

Usually, correlations are investigated between clinical features and TMS measures recorded at rest. However, resting-state physiology may not be representative of alterations occurring during symptom expression. For example, CSE measures at rest in patients with parkinsonism may not be applicable to bradykinesia, which occurs during movement. A more appropriate assessment of how corticospinal physiology relates to bradykinesia would be assessing it during repetitive, continuous movements, when bradykinesia manifests. A corollary in AD would be to employ temporo-parietal TMS during behavioral tasks of memory recall; note that this would require an additional neuroimaging tool such as concurrent EEG to measure the output of TMS. In all, the investigation of symptoms in neurodegenerative diseases should aim toward one where the symptom is expressed and hence the network driving said symptom is assessed. Casula et al., for example, combined TMS-EEG with a speeded tapping task to assess how changes in cortical oscillatory activity are related to impairments in movement generation in Huntington's disease (HD). They found that stronger oscillatory activity corresponded to better performance (240), showing that the timing accuracy of cortical synchronization and desynchronisation may be a physiological basis for clinical features of HD.

## Conclusion

TMS is a powerful tool to investigate corticospinal and cortical physiology *in vivo*. This review has summarized how TMS has given valuable insights into the pathophysiology of neurodegenerative diseases. We made several recommendations for how TMS should be employed in the future to better inform the pathophysiology of neurodegenerative diseases. The usefulness of TMS has traditionally been limited by the lack of an objective output and hence has been confined to stimulation of M1; this limitation can be overcome by the use of concurrent neuroimaging methods such as EEG. Seeing as neurodegenerative diseases evolve over time, TMS measures should aim to track longitudinal changes, especially when the aim of the study is to look at disease progression and symptomatology. Although TMS measures have been shown to aid in the diagnosis of neurodegenerative diseases, the lack of gold-standard diagnostic confirmation undermines the validity of the findings. Consequently, diagnostic certainty should be maximized through a variety of methods including multiple, independent clinical assessments, imaging and fluids biomarkers and post-mortem pathological confirmation where possible. There is great interest in understanding the mechanisms by which symptoms arise in neurodegenerative disorders. However, TMS assessments in patients are usually carried out during resting conditions, when the brain network engaged during these symptoms is not expressed. Rather, a context-appropriate form of TMS would be more suitable in probing the physiology driving clinical symptoms. In all, we hope that the recommendations made here will help to further understand the pathophysiology of neurodegenerative diseases.

## Author Contributions

VR, AL, and LR wrote the manuscript. NS and JR provided valuable insights in the direction of the manuscript. All authors contributed to the article and approved the submitted version.

## Conflict of Interest

The authors declare that the research was conducted in the absence of any commercial or financial relationships that could be construed as a potential conflict of interest.
